# The extended TILAR approach: a novel tool for dynamic modeling of the transcription factor network regulating the adaption to *in vitro* cultivation of murine hepatocytes

**DOI:** 10.1186/1752-0509-6-147

**Published:** 2012-11-29

**Authors:** Sebastian Vlaic, Wolfgang Schmidt-Heck, Madlen Matz-Soja, Eugenia Marbach, Jörg Linde, Anke Meyer-Baese, Sebastian Zellmer, Reinhard Guthke, Rolf Gebhardt

**Affiliations:** 1, Leibniz Institute for Natural Product Research and Infection Biology - Hans-Knöll-Institute, Beutenbergstr. 11a, D-07745 Jena, Germany; 2 Institute for Biochemistry, Faculty of Medicine, University of Leipzig, Johannesallee 30, D-04103 Leipzig, Germany; 3 Department of Scientific Computing, Florida State University, Florida 32310-4120, Tallahassee, USA; 4, GermanFederal Institute for Risk Assessment, Max-Dohrn Str. 8-10, D-10589 Berlin, Germany

**Keywords:** Gene regulation, Dynamic network inference, Transcription factor networks, Key regulator identification, Linear modeling, Least angle regression, Hepatocytes, Liver, Atf3 - activating transcription factor 3, Dbp - D site albumin promoter binding protein, Tgif1 - TGFB-induced factor homeobox 1

## Abstract

**Background:**

Network inference is an important tool to reveal the underlying interactions of biological systems. In the liver, a complex system of transcription factors is active to distribute signals and induce the cellular response following extracellular stimuli. Plenty of information is available about single transcription factors important for the different functions of the liver, but little is known about their causal relations to each other.

**Results:**

Given a DNA microarray time series dataset of collagen monolayers cultured murine hepatocytes, we identified 22 differentially expressed genes for which the corresponding protein is known to exhibit transcription factor activity. We developed the Extended TILAR (ExTILAR) network inference algorithm based on the modeling concept of the previously published TILAR algorithm. Using ExTILAR, we inferred a transcription factor network based on gene expression data which puts these important genes into a functional context. This way, we identified a previously unknown relationship between Tgif1 and Atf3 which we validated experimentally. Beside its known role in metabolic processes, this extends the knowledge about Tgif1 in hepatocytes towards a possible influence of processes such as proliferation and cell cycle. Moreover, two positive (i.e. double negative) regulatory loops were predicted that could give rise to bistable behavior. We further evaluated the performance of ExTILAR by systematic inference of an *in silico* network.

**Conclusions:**

We present the ExTILAR algorithm, which combines the advantages of the regression based inference algorithm TILAR, like large network sizes processable and low computational costs, with the advantages of dynamic network models based on ordinary differential equation (i.e. *in silico* knock-down simulations). Like TILAR, ExTILAR makes use of various prior-knowledge types such as transcription factor binding site information and gene interaction knowledge to infer biologically meaningful gene regulatory networks. Therefore, ExTILAR is especially useful when a large number of genes is modeled using a small number of experimental data points.

## Background

One of the aims in systems biology is to reveal functions and uncover causalities in the behavior of biological systems. As these systems are usually a composition of multiple processes, mathematical modeling is often applied to investigate processes of interest. The understanding of the parts contributes to the understanding of the system as a whole. One biological process of interest is the regulation of gene expression which is mostly influenced by transcription factors (TFs). These regulating proteins can have an activating or repressing effect on the expression of a gene. The extend of regulation largely depends on the activity of the TF which is determined on multiple levels, mostly the post-translational level [1]. Therefore, the gene expression profile of a TF can generally not be considered as its activity profile. However, the target genes, their regulators (TFs) and the relations between these entities constitute a gene regulatory network (GRN) which gives information about the functions of the individual genes. This network is commonly represented as a graph where nodes correspond to the genes and edges are the regulatory relations between them.

To reconstruct GRNs, gene expression data-based network inference is a widely accepted approach. Although high-throughput technologies such as microarrays and RNA-seq have become more accessible (in terms of quality of the measurements, decreasing costs and advancing standard operating procedures) there are still central problems that hamper their inference. A major difficulty is that the number of available measurements is usually lower than required. Often, more genes than the number of available measurements are included in the model. This leads to an underdetermined system with a large amount of possible solutions. When dealing with time series data, a low temporal resolution of measurements contributes to this problem making it more difficult to obtain a reliable solution. Additionally, the usually low number of replicates does not account for the variability introduced by the methods of measurement and the natural biological variation. Hecker *et al.*[2] highlighted the relationship between the complexity of the model, the data required to explain the observed behavior and the quality of the inference result. Different algorithms have been proposed that cope with the aforementioned problems in various ways. For reviews on the different approaches see [2,3]. Depending on the purpose of the model, we can distinguish these approaches by splitting them into two groups, algorithms that produce models that are able to quantitatively describe the dynamic behavior of the network, and algorithms which do not. Algorithms that belong to the first group are usually based on difference or differential equations. Although these models offer advantages such as the simulation of the dynamics and the modeling of complex relations between the components of a network, there are also drawbacks like an increased computational effort for network structure and parameter optimization. As a consequence, the number of genes that can be modeled is limited. Therefore, pre-selection of genes is required which supposes a vast prior-knowledge about the relevant genes and processes. One of the freely accessible, ready-to-use algorithms that falls into this category is the successfully applied [4-6] Net*Gene*rator algorithm [7,8].

Depending on whether or not the utilized model is based on linear or non-linear differential equations the number of free parameters and therefore the detail of the model, but also the complexity of the inference problem is increasing drastically [2]. There is always a trade off between the simplification of the real biological system under observation and the loss of important mechanisms of regulation [9]. The high degree of detail enables non-linear models to represent the dynamic behavior of biological and biochemical systems in an adequate manner. Due to the high number of free parameters however, these models are often used under presumptions such as the availability of large amounts of data [10], a known network structure, constrains regarding the network structure [11,12], or additional kinetic knowledge [13] or assumptions (such as nonlinear sigmoidal activation functions as used by Mjolsness *et al.*[14] or Toepfer *et al.*[8]). A lack of these presumptions may force modelers to choose linear over non-linear models to minimize the number of parameters to estimate and reduce the search space. However, despite the loss of detail linear models have been shown to successfully represent regulatory networks that are able to effectively uncover causal relations between the entities of biological processes such as in [5,15].

In the second group of algorithms, there is a trade-off between the flexibility and possibility for quantitative, dynamic modeling and the advantage of processing larger network sizes. There are regression-based algorithms such as LASSO [16], Least Angle RegreSsion (LARS) [17] or Transcription Factor binding site integrating LARS (TILAR) [18], correlation-based algorithms [19] and information theory-based algorithms such as the Algorithm for the Reverse engineering of Accurate Cellular Networks (ARACNE) [20], MRNET [21] or Context Likelihood of Relatedness (CLR) [22], which are able to infer large networks. Regression based algorithms were successfully used to infer full genomic networks [23,24]. These algorithms use simple models and are known to be fast. Some of the methods were also shown to construct networks that tend to fulfill structural properties such as scale-freeness [25], which has been observed in real, existing biological networks [26]. Furthermore, the ARACNE algorithm was extended to the Time-Delay ARACNE (TD-ARACNE) [27] algorithm, which is able to consider temporal information.

In the following, we present an algorithm which combines the advantages of both of these classes, fast inference of medium size networks that can quantitatively model the dynamic behavior of the inferred network. We extend the existing TILAR algorithm that uses a linear network model based on the LARS algorithm. Networks inferred with TILAR consist of two types of nodes, the genes with measured expression profile to model and the regulating TFs, that connect these genes. Due to this concept of modeling, the algorithm makes use of various biological knowledge sources such as transcription factor binding site (TFBS) information and gene interaction knowledge. This information decreases the number of possible network structures and therefore, allows fast inference of reliable, biologically meaningful networks. While the TFBS knowledge is represented by the network edges that go from a regulating TF to a target gene (TF-to-gene relations), the gene interaction knowledge is represented by the edges that connect the target genes with the regulating TFs (gene-to-TF relations).

Extended TILAR (ExTILAR) adapts this modeling concept to produce network models that are based on linear ordinary differential equations. This allows the inference of networks from time series data, which can be used to uncover the most important unknown relations between genes and to identify potential key regulators. Linear models represent approximations close to a steady state (operating point) of non-linear models that are adequate for living systems in principle. Non-linear terms can and should be included in the proposed modeling algorithm if prior knowledge about the type of non-linearity is available and if the number of experimental data is sufficient to identify the increased number of model parameters. However, the automatic identification of additional non-linear model terms in general requires more independent experimental data in order to ensure a stable convergence of the algorithm to a unique model structure (see in [2] section 3.3.2). ExTILAR makes use of all replicate-measurements at once, which produces stable networks that are robust to small variations in the data. To assess the performance of ExTILAR, the algorithm was applied to *in silico* data. The results were compared to those obtained by the published network inference tool Net*Gene*rator [7,8].

ExTILAR was applied to data from Zellmer *et al.*[28] which monitors the response of murine primary hepatocytes to the exchange of culture medium after a period of starvation. We investigated a set of differentially expressed genes for which the corresponding proteins are known to exhibit transcription factor activity (DETF). For some of these DETFs, little about their function in hepatocytes or liver in general was found in literature. By inferring a transcription factor network (TFN) (a GRN consisting of only TFs) with ExTILAR using the extracted DETFs, we study their potential roles in the cellular response and identify new causal relations. Subsequently, processing of the data and knowledge extraction will be described. Consecutively, the modeling concept of TILAR will be outlined, followed by the introduction of ExTILAR and a detailed description of its modified modeling concept. Finally, the results of the inference will be presented and analyzed. For validation, a knock-down experiment was performed which confirmed the predicted relation between the TFs Tgif1 and Atf3, and Dbp and Atf3.

## Results and discussion

### Inference from biological data

**Figure 1:**
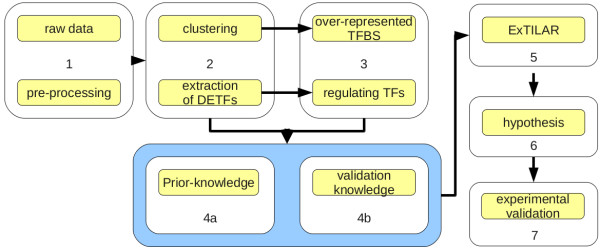
**Workflow used to analyze the response of murine primary hepatocytes to the change of culture medium after a period of starvation.** The workflow of the ExTILAR inference study presented here can be roughly divided into 7 single steps. After pre-processing of the the raw data (step 1), the gene expression profiles were clustered and DETFs were extracted (step 2). Over-represented TFBSs for the clusters were determined. Regulating TFs for the selected DETFs were extracted from literature knowledge (step 3). In step 4, the information of the two previous steps were pooled to extract prior-knowledge and validation knowledge. Using the expression profiles of the DETFs, the mean cluster expression profiles, the information about the regulating TFs and the prior-knowledge, ExTILAR was applied to infer a TFN (step 5). The resulting network was checked for present validation knowledge, analyzed and interpreted to extract testable hypothesis (step 6). The extracted hypotheses were validated experimentally (step 7).

Data of primary murine hepatocytes from Zellmer *et al.*[28] were used to investigate the cellular response to the change of culture medium after a period of starvation (24 hours). Originally, a time series experiment (t = 3, 24, 27, 30, 36, 48 hours after isolation) with 2 biological replicates at the first time point and 3 biological replicates at the remaining time points was performed using 17 Affymetrix MOE4302 microarrays. For this study, we investigated only the second phase of the experiment (24 hours to 48 hours). Therefore, the time points will be subsequently referred to as 0, 3, 6, 12 and 24 hours after the change of culture medium. The workflow consists of 7 steps (Figure 1), data pre-processing, identification of differentially expressed genes, clustering and extraction of DETFs (genes included in the model), extraction of regulatory relations between TFs and the DETFs (TF-to-gene relations), extraction of prior-knowledge for the DETFs and the TFs (gene-to-TF relations), the network inference, the interpretation and analysis of the inferred TFN and finally, experimental validation of the extracted hypotheses.

#### Pre-processing and gene filtering

The latest custom chip definition file from Brainarray [29] (version 15) based on Entrez gene ID’s was used to annotate the microarrays. Pre-processing was performed using the standard robust multi-array average (RMA) [30,31] procedure. Detection calls [32] were calculated and used for filtering of probe sets (see Methods). This resulted in 6306 genes that were analyzed for differential expression using a 2-fold-change criterion. This resulted in 950 identified differentially expressed genes (DEGs) (Additional file 1). The enrichment analysis using GOstats [33] showed that the DEGs are associated with various metabolic processes such as organic acid metabolism, steroid and lipid metabolic processes (Additional file 2).

#### Clustering

**Figure 2:**
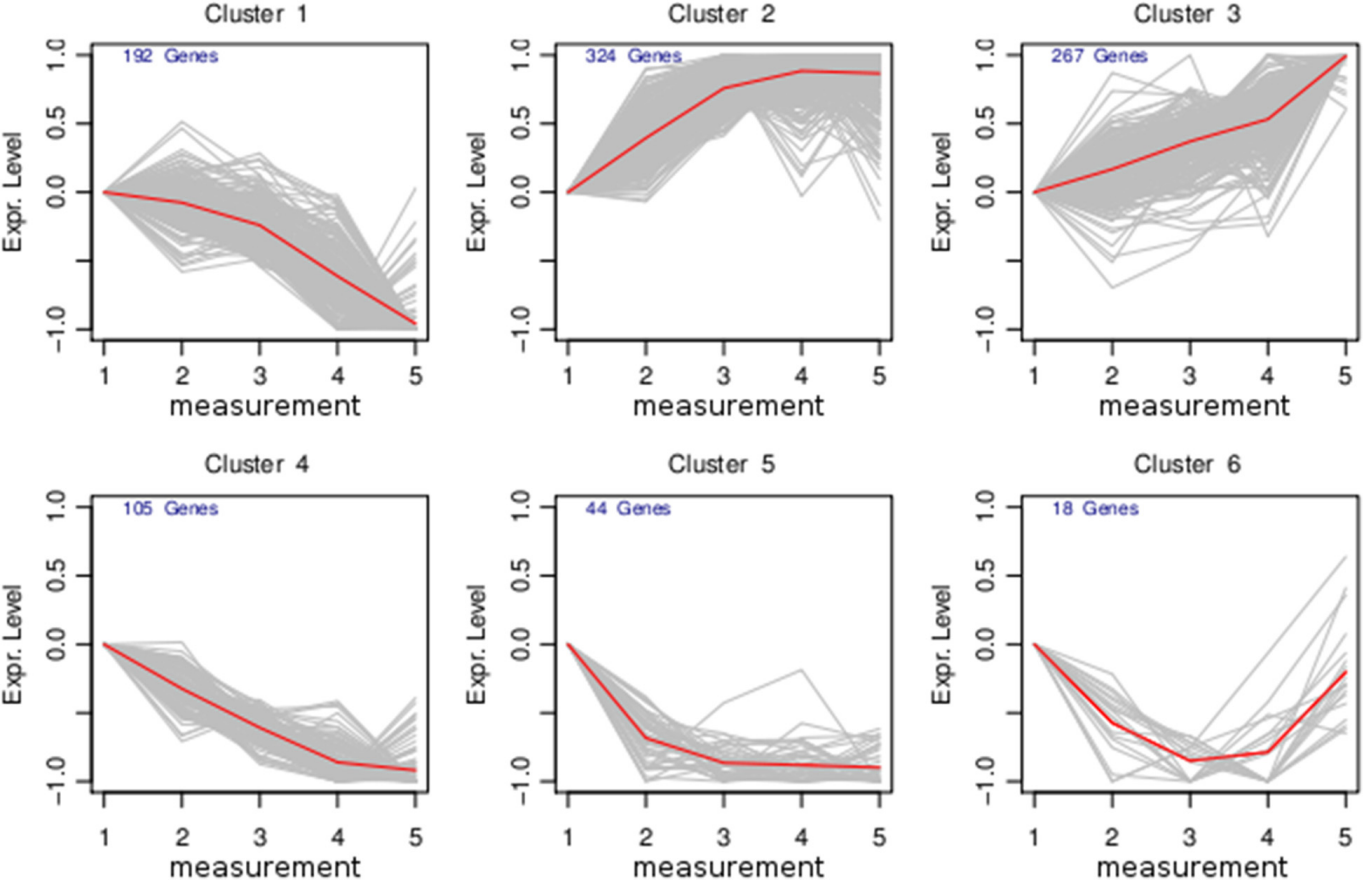
**Results of the SOTA clustering.** Clustering of the 950 differentially expressed genes resulted in 6 clusters denoted as slow down (cluster 1), fast up (cluster 2), up (cluster 3), down (cluster 4), fast down (cluster 5) and middle peak down (cluster 6). Shown are the median scaled log2-FC expression profiles (’Expr.Level’). The results of the enrichment analysis for each cluster are outlined in the Additional file 3.

To identify groups of similarly regulated genes, the DEGs were clustered according to their expression profile using the self-organizing tree algorithm (SOTA) [34,35]. This resulted in six clusters denoted as up, slow-up, fast-down, down, slow-down and middle-peak-down (Figure 2). GOstats was used to perform an enrichment analysis for each cluster (Additional file 3). The results show an increasing tendency over time in the expression profile of genes associated with oxidation-reduction processes and glutathione metabolic processes (cluster 3), translation activity and the ribosome (cluster 2). Genes associated with other metabolic processes such as the fructose, glucose, lipid and steroid metabolic process (cluster 1 and 4) decreased in their expression level over time.

#### Extraction of DETFs

A total of 22 DETFs were extracted by filtering all DEGs associated with the GO-category “sequence-specific DNA binding transcription factor activity” (GO:0003700). According to the Gene Ontology (GO) terms obtained from the MGI database [36], it was found that almost all extracted DETFs can be connected to either metabolic processes or differentiation/cell faith processes (Table 1).

**Table 1:** Detailed information about the DETFs

**DETF**	**Cluster**	**Associated biological processes**
Atf3	5	gluconeogenesis; regulation of cell proliferation
Cebpa	4	liver development; fat cell differentiation; regulation of cell proliferation; urea cycle
Cebpb	6	cell differentiation; anti-apoptotic
Cebpd	4	fat cell differentiation
Csrnp1	5	apoptotic process; platelet-derived growth factor receptor signaling pathway
Dbp	6	rhythmic processes
E2f6	2	regulation of transcription involved in G1/S phase of mitotic cell cycles;
Egr1	6	BMP signaling; Il1 mediated signaling pathway; regulation of Wnt signaling pathway; regulation of cell-death; response to glucose stimulus; response to insulin stimulus
Fos	6	cellular response to extracellular stimuli; response to stress
Foxa1	2	glucose homeostasis; chromatin remodeling
Gatad1	2	-
Id3	3	regulation of cell cycle; regulation of apoptosis
Irf1	3	cellular response to Il1; regulation of cell-death
Klf16	6	-
Maff	5	epidermal cell-differentiation
Nr1h4	2	bile acid metabolic process; regulation of carbohydrate- and urea metabolic process;
Ppara	4	Glucose metabolic process; lipid metabolic process; response to insulin stimulus
Srebf1	4	Steroid metabolic process; response to glucose stimulus; lipid metabolic process;
Srf	4	actin filament organization; cell-cell adhesion; developmental growth;
Tgif1	3	regulation of cell proliferation; regulation of retinoic acid receptor signaling pathway
Tsc22d1	2	regulation of apoptotic process; regulation of cell proliferation
Zbtb16	4	positive regulation of apoptosis; negative regulation of proliferation

DETF cluster membership and associated biological functions based on the information of GeneOntology. For all but Gatad1 and Klf16 it was found that the TFs can either be associated with metabolic processes and/or cell faith.

#### Extraction of prior-knowledge

oPOSSUM was used to identify possible regulators for each cluster [37,38]. Therefore, the promoter region from the transcription start site to 2000bp upstream was used to find over-represented TFBSs using the binding site information from the Jaspar database [39]. This resulted in a total of 79 TFs for the six clusters.

Transfac [40] and Pathway Studio 8.0 [41] were used to identify regulating TFs that are known to have an influence on the expression of the extracted DETFs of the data set (TF-to-gene relations). While Transfac contains experimentally validated TFBS for most of the identified TFs, Pathway Studio was applied to extend the list of potential regulators using literature knowledge derived by text-mining. This way, 215 TF-to-gene relations were extracted for 16 DETFs. However, no TFBS information was retrieved for six DETFs (Gatad1, Csrnp1, Dbp, Klf16, Maff and Tsc22d1) using either of the two approaches. To make them available for the network inference process, an artificial TF was added for each of them. This is necessary as the modeling concept of TILAR-based algorithms allows to model gene regulation exclusively via TFs and not directly between the genes. Each DETF should have at least one TF to act not only as the regulating source, but to be also available as a target of regulation. This resulted in a total of 323 TF-to-gene relations for the clusters as well as for the DETFs. prior-knowledge was obtained using Pathway Studio 8. 543 gene-to-TF relations were extracted where a DETF was identified to modulate the expression of a potentially regulating TF.

#### Extraction of validation knowledge

PathwayStudio 8.0 was used to extract known, direct relations between the DETFs included in the model. As these 36 relations are not used for the network inference process, they can be used to validate the final network.

### TFBS integrating LARS (TILAR)

**Figure 3:**
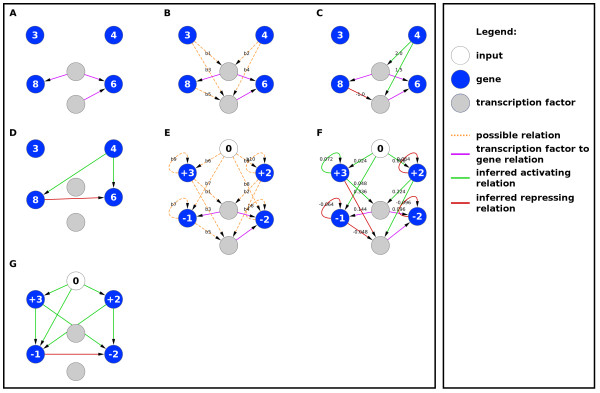
**Concept of modeling of TILAR (A-D) compared to ExTILAR (E-G). A-D**) The modeling concept of the TILAR algorithm. The genes are labeled with their expression values. **A**) In TILAR, a gene can only be regulated by another gene via a TF *k* if the regulating gene does not possess a TFBS for the TF *k* itself (TF-to-gene realtions). **B**) This decreases the number of possible network topologies and therefore serves as a additional source of prior-knowledge (gene-to-TF relations). **C**) LARS is used to infer a sparse network which explains the measured expression values of the genes in the best possible way. A constrained ordinary least square (OLS) approach is used to estimate the parameters using the final structure obtained from LARS. **D**) This way, new hypotheses about gene to gene relations can be obtained. **E-G**) The extended concept of modeling used by ExTILAR. Since the algorithm estimates the change of expression of each gene over time, the nodes are labeled with ${ŷ}^{i}=\frac{\Delta x_{i}}{\mathrm{\Delta t}}$ where *Δx*_*i*_=*x*_*i*_[*t*_*m*_]−*x*_*i*_[*t*_*m*−1_] and *Δt*=1 is outlined in the labels of the corresponding genes. **E**) The number of possible network structures is lowered by the TFBS information. Additionally, auto-regulation and modeling input perturbations are introduced and increase the number of regression coefficients to estimate. **F**) One possible model is selected from the full set of models returned by LARS. A OLS approach is used to find the parameters, given the network structure of the selected model. **G**) The gene expression dynamics of the final network can be simulated using standard ODE-solvers.

TILAR uses a linear network model to construct GRNs based on LARS [18]. A particular feature of the method is the ability to integrate multiple sources of knowledge into the inference process, namely TFBS information (TF-to-gene relations) as well as text-mining knowledge about the regulation of TFs by the genes (gene-to-TF relations). TILAR prohibits the direct interaction of genes but allows interaction via TFs. In that, a regulating gene modulates the activity of a TF, which in turn regulates the expression of the target gene (Figure 3A-D). This concept is more biologically realistic than concepts that rely only on direct gene-to-gene interactions and copes with the known problem that the activity of transcription factors is not only regulated on the transcriptional level but also through various other mechanisms such as (de)phosphorylation and dimerization [1]. Based on expression data only, the modeling of TF activity can lead to falsely inferred interactions. Therefore, TILAR does not model the TF activity but uses the TFBS knowledge to model them as “bridges” between the genes. This way, regulating TFs are included in the model but no additional information such as expression or activity profiles have to be known. Additionally, the number of possible network structures is reduced as multiple genes can share the same TF. This concept can be expressed in the following equation:

(1) $$\begin{array}{c} \;{\hat{x}}_{i}=\overset{F}{\underset{k=1}{\sum }}\overset{N}{\underset{j=1}{\sum }}(1-b_{\mathrm{kj}})w_{\mathrm{kj}}x_{j}b_{\mathrm{ki}}\\ \;\text{with}\\ b_{\mathrm{kj}}=\left\{\begin{array}{c} \;1,\;\;\text{if gene}j\text{possesses a binding site for TF}k\\ \;0,\;\;\text{else} \end{array}\right) \end{array}$$

The predicted expression level ${\hat{x}}_{i}$ of the regulated gene *i* is the result of the sum of the weighted expression levels *w*_*kj*_*x*_*j*_ of all regulating genes *j* (*j*=1…*N*) via the transcription factor *k* (*k*=1…*F*) if (i) the gene *i* has a binding site for the TF *k* and (ii) the gene *j* is not regulated by the TF *k*.

To use regression for the estimation of the parameters *w*_*kj*_, equation 1 has to be expressed in the basic regression model form $ŷ=X_{M\times N}*\beta$ where ŷ is the prediction vector that contains the predicted values corresponding to the observed values in the response vector *y*=*x*, *β* denotes for the parameters *w*_*kj*_and *X* corresponds to the regression matrix that contains the observed measurements *x*_*ij*_ (with *i*=1,…,*N* and *j*=1,…,*M*) where *N* is the number of variables and *M* is the number of measurements. Therefore, given a gene *i* (*i*=1,…,*N*) which possesses at least one TFBS, the equation 1 can be expressed in matrix form:

$$\;\begin{array}{c} \;{ŷ}^{i}=X_{M\times \left(\mathrm{NF}-B^{i}\right)}^{i}*\beta^{i}\;\text{for}\;i=1\ldots N\;\text{, where}\\ \text{(i)}\;{ŷ}^{i}=x_{i}\\ \text{(ii)}\;X_{M\times \left(\mathrm{NF}-B^{i}\right)}^{i}=\left(x_{1},\ldots ,x_{j},\ldots ,x_{\left(\mathrm{NF}-B^{i}\right)}\right)\\ \;\text{with}\;\;x_{j}={\left(x_{1j},\ldots ,x_{\mathrm{Mj}}\right)}^{T}\; \end{array}$$

(2) $$\;\begin{array}{cc} \text{(iii)} & \begin{array}{cc} \beta^{i}={\left(\beta^{1},\ldots ,\beta^{k},\ldots ,\beta^{F}\right)}^{T} & \;\forall k\text{that can bind to}i\;\;\text{with}\\ \;\;\beta^{k}=\left(w_{k1},\ldots ,w_{\mathrm{kj}},\ldots ,w_{\mathrm{kN}}\right) & \;\forall j\text{that do not possess}\\ \;\text{a TFBS for}k \end{array} \end{array}$$

The predicted expression value ${ŷ}^{i}$ of gene *i* is calculated using the vector of regression coefficients *β*^*i*^ and the regression matrix *X*^*i*^ which contains the observed expression values *x*_*j*_of the genes *j* (*j*=1…*N*). *X*^*i*^ is composed of *M* rows and *NF*−*B*^*i*^ columns, where *M* is the number of measurements and *B*^*i*^denotes for the number of TF *k* to gene relations where (i) the TF *k* is not regulating the gene *i* or (ii) the TF *k* is regulating gene *j* or both. To estimate all parameters at once, the equations for the *N* genes can be jointly expressed in matrix form:

(3) $$\;\begin{array}{c} \;ŷ=X_{M^{\prime }\times N^{\prime }}*\beta \\ \text{with}\;M^{\prime }=MN_{r}\;\text{and}\;N^{\prime }=\mathrm{FN}-B\;\text{, where}\\ \text{(i)}\;ŷ=x\\ \text{(ii)}X_{M^{\prime }\times N^{\prime }}\;=\;(x_{1},\ldots ,x_{j},\ldots ,x_{N^{\prime }})\text{with}\;\;x_{j}\;=\;{\left(x_{1j},\ldots ,x_{M^{\prime }j}\right)}^{T}\\ \;\beta ={\left(\beta^{1},\ldots ,\beta^{k},\ldots ,\beta^{F}\right)}^{T}\text{with}\\ \text{(iii)}\beta^{k}=\left(w_{k1},\ldots ,w_{\mathrm{kj}},\ldots ,w_{\mathrm{kN}}\right)\;\forall j\;\text{that do not possess}\\ \;\text{a TFBS for}k \end{array}$$

The regression matrix *X* is composed of *MN*_*r*_rows and *FN*−*B*columns where *N*_*r*_is the number of genes that possess at least one transcription factor binding site and *B* denotes for the number of TF-to-gene relations.

Variable selection and estimation of the regression coefficients can be performed by using the least shrinkage and selection operator (LASSO) algorithm, a constraint ordinary least square (OLS) approach [16]. Selecting a candidate vector of regression coefficients $\hat{\beta }={\left({\hat{\beta }}_{1},\ldots ,{\hat{\beta }}_{N^{{}^{\prime }}}\right)}^{T}$ (with $\hat{\beta }\in \hat{B}$) of the set of all possible candidate regression coefficient vectors $\hat{B}$, we calculate the prediction vector ${ŷ}^{i}$ (with *y*^*i*^=*x*_*i*_and *i*=(1,…,*M*^*′*^):

(4) $$\begin{array}{c} \;\;{ŷ}^{i}=\overset{N^{\prime }}{\underset{j=1}{\sum }}x_{\mathrm{ij}}{\hat{\beta }}_{j}\;\text{with}\\ X_{M^{{}^{\prime }}\times \;N^{\prime }}={\left(x_{1},\ldots ,x_{i},\ldots ,x_{M^{\prime }}\right)}^{T}\text{and}\\ \;x_{i}=\left(x_{i1},\ldots ,x_{\mathrm{ij}},\ldots ,x_{iN^{\prime }}\right) \end{array}$$

with the residual sum of squares (RSS)

(5) $$\mathrm{RSS}(\hat{\beta })=\overset{M^{\prime }}{\underset{i=1}{\sum }}{\left(y^{i}-{ŷ}^{i}\right)}^{2}$$

LASSO chooses the vector of regression coefficients ${\hat{\beta }}^{*}$ which minimizes the RSS

(6) $${\hat{\beta }}^{*}={\arg\min}_{\hat{\beta }}\mathrm{RSS}(\hat{\beta })$$

with the additional constrain

(7) $$\overset{N^{\prime }}{\underset{j=1}{\sum }}\delta_{j}|{\hat{\beta }}_{j}|\leq s$$

that the sum of the absolute regression coefficients is lower than a certain threshold *s* (equation 7). This controls the sparseness of the resulting model. When using the adaptive LASSO approach, an additional weighting parameter *δ*_*j*_(*j*=1…*N*^*′*^) is specified (within the range of [0,1]) to shrink the regression coefficient ${\hat{\beta }}_{j}$ and thus, support the insertion of the corresponding prior-knowledge gene-to-TF edge into the model. The modified Least Angle Regression (LARS) algorithm was shown to produce the full set of the LASSO estimates with an increased computational efficiency [17]. Therefore, the adaptive LARS is used instead of the adaptive LASSO.

### Extended TILAR (ExTILAR)

TILAR was extended to enable the inference of gene regulatory networks from time resolved data by a system of differential equations approximated by a set of difference equations with the time interval *Δt*=*t*_*m*_−*t*_*m*−1_. By approaching *Δt*towards zero, the difference equations become differential equations which can be numerically solved with standard algorithms. In ExTILAR, the possibility to model the systems response to external perturbations as well as the possibility of auto-regulation were added to the modeling approach of TILAR. It is important to note that the auto-regulation term as it is used in the model does capture the sum of all effects that might influence the abundance of the transcript such as self-regulation and RNA-degradation. In that, a gene may be regulated by (i) other genes via TFs, (ii) the sum of auto-regulatory effects and (iii) the input of one or more external perturbations (Figure 3E-G). Thus, the parameters to estimate are assigned to gene-to-TF interactions, auto-regulation and input signal to gene interactions. The modeling concept of ExTILAR can be expressed as:

(8) $$\begin{array}{c} \frac{x_{i}\left[t_{m}\right]-x_{i}\left[t_{m-1}\right]}{t_{m}-t_{m-1}}=G(i,m)+A(i,m)+I(i,m)\\ G(i,m)\;\;\;\;=\overset{F}{\underset{k=1}{\sum }}\overset{N}{\underset{j=1}{\sum }}\left(1-b_{\mathrm{kj}}\right)w_{\mathrm{kj}}x_{j}\left[t_{m-1}\right]b_{\mathrm{ki}}\\ A(i,m)\;\;\;\;=a_{i}x_{i}\left[t_{m-1}\right]\\ I(i,m)\;=\overset{C}{\underset{c=1}{\sum }}d_{\mathrm{ci}}u_{c}\left[t_{m-1}\right]\\ \;\text{with}\\ b_{\mathrm{kj}}\;=\left\{\begin{array}{c} 1\;\text{, if gene}j\text{possesses a}\\ \;\text{binding site for TF}k\\ 0\;\text{, else} \end{array}\right) \end{array}$$

According to this equation, the quotient of difference of expression $\frac{\Delta x_{i}}{\mathrm{\Delta t}}=\frac{x_{i}\left[t_{m}\right]-x_{i}\left[t_{m-1}\right]}{t_{m}-t_{m-1}}$ of the gene *i* (*i*=1…*N*) from the time point *t*_*m*−1_ to *t*_*m*_ is the sum of three terms. The first term (*G*(*i*,*m*)) describes the weighted influence *w*_*kj*_ of the regulatory genes *j* (*j*=1…*N*) at *t*_*m*−1_ on the expression level *x*_*i*_of gene *i* at *t*_*m*−1_ via the TF *k* (*k*=1…*F*). The gene *i* is regulated by the genes *j* via the TF *k* if, (i) *i* possesses a TFBS for *k* and (ii) *j* is not regulated by *k*. This term equals the original TILAR model shown in equation 1. The second term (*A*(*i*,*m*)) describes the auto-regulatory effect *a*_*i*_at the expression level *x*_*i*_ of gene *i* at *t*_*m*−1_. The third term (*I*(*i*,*m*)) describes the influence of the input perturbation *u*_*c*_(*c*=1…*N*) at *t*_*m*−1_. As outlined in the TILAR section, equation 8 has to be expressed in regression model form to allow parameter estimation using LARS in the aforementioned way. The full set of equations for all genes is a N-coupled system and can be expressed in matrix form:

(9) $$\;\begin{array}{c} \;\;\;\;\;\;ŷ=X_{M^{\prime \prime }\times N^{\prime \prime }}*\beta \;\text{with}\\ M^{\prime \prime }=(M-1)N_{r}^{\prime }R\;\text{and}\;N^{\prime \prime }=N^{\prime }+U+A,\;\;\text{where}\\ \text{(i)}\;ŷ=\frac{\mathrm{\Delta x}}{\mathrm{\Delta t}},\\ \text{(ii)}\;X_{M^{\prime \prime }\times N^{\prime \prime }}=\\ \;\;\;\;\left(x_{1},\;\ldots ,x_{j},\ldots ,x_{N^{\prime }},u_{1},\ldots ,u_{l},\ldots ,u_{U},x_{1},\ldots ,x_{h},\ldots ,x_{A}\;\right)\\ \;\;\;\;\;\;\text{with}\\ \;\;\;\;x_{j}={\left(x_{1j},\ldots ,x_{M^{\prime \prime }j}\right)}^{T}\\ \;\;\;\;u_{l}=\left(u_{1l},\ldots ,u_{M^{\prime \prime }l}\right)\\ \;\;\;\;x_{h}=\left(x_{1h},\ldots ,x_{M^{\prime \prime }h}\right),\\ \text{(iii)}\beta \;=\;{\left(\;\beta^{1}\;,\ldots ,\beta^{k}\;,\ldots ,\beta^{F}\;,\beta^{1}\;,\ldots ,\beta^{c},\ldots ,\beta^{C},a_{1},\ldots ,a_{A}\;\right)}^{T}\\ \;\;\;\;\;\;\text{with}\\ \begin{array}{cc} \beta^{k}={\left(w_{k1},\ldots ,w_{\mathrm{kj}},\ldots ,w_{\mathrm{kN}}\right)}^{T} & \begin{array}{c} \;\forall j\text{that do not possess}\\ \text{a TFBS for}k\text{,}\; \end{array}\\ \beta^{c}=\left(d_{c1},\ldots ,d_{\mathrm{cj}},\ldots ,d_{\mathrm{cU}}\right) & \begin{array}{c} \forall j\text{that can be}\\ \text{regulated by}c \end{array} \end{array} \end{array}$$

Here, the regression matrix *X* is composed of $\left(M^{\prime \prime }=(M-1)N_{r}^{\prime }R\right)$ rows and *N*^*′′*^=(*N*^*′*^ + *U* + *A*) columns. $\left(N_{r}^{\prime }\right)$ denotes for the number of genes which have at least one TFBS (*N*_*r*_) or at least one input to gene relation. Because temporal information is considered in equation 8, given an experiment with *M* time points, we calculate *y* as the quotient of difference of expression $\frac{\mathrm{\Delta x}}{\mathrm{\Delta t}}$, which leaves us with *M*−1 measurements for each gene *i* ( $\left(i=1,\ldots ,N_{r}^{\prime }\right)$).

Experiments often have biological replicates for the measurements at each time point. This leads to the same time series being measured *R* times. As ExTILAR makes use of these replicates by including them in the regression matrix *X*, there is a total of *M*−1 measurements for each gene *i* for each of the time-series replicates *R*. Compared to equation 3, *U* + *A*columns are added to the regression matrix *X* where *U* is the number of input-to-gene relations and *A* denotes for the number of genes which are auto-regulated. Since only genes, which possess at least one TFBS or at least one input-to-gene relation are considered in the rows of *X*, *A* equals $\left(N_{r}^{\prime }\right)$.

LARS can now be used to efficiently perform automatic variable selection and simultaneous regression coefficient estimation (equations 4-7). However, for the adaptive LARS it is important to notice that the *δ*_*j*_parameter in equation 7 is now determining the integration of the gene-to-TF prior-knowledge edges, as well as the input-to-gene edges and the auto-regulatory edges. This way, not only prior-knowledge but also auto-regulation as well as regulation by the input can be soft-integrated into the model. The *δ*parameter can be set between one and zero with increased integration for values close to zero.

Although the introduction of these input parameters offers greater possibilities for fine-tuning of the algorithm, parameter identification is a crucial step for the inference of networks from biological data. A major problem is that the true underlying structures and processes are often unknown and the mathematical model that the inference algorithm is based on can always only be an abstraction of the truth. Therefore, a good set of parameters needs to be found which leads to inferred networks that maximize the amount of integrated biological prior-knowledge and adequately reproduce the observed dynamics. Regarding the integration of prior-knowledge a requirement is that the knowledge used to infer the network must be different from the knowledge that is used for its validation. An advantage of the TILAR-family algorithms is that gene-to-TF knowledge is used during the inference, which can be obtained by literature text-mining. The gene-to-TF relations together with the TF-to-gene relations implicitly define gene-to-gene interaction information which can also be derived from literature. Therefore, this concept of modeling makes use of two distinct prior-knowledge sets, one that is used during the network inference and one that is used for validation purposes only. The advantage of using these multiple prior-knowledge sources was shown by Hecker *et al.*[2]. Comparing the TILAR inferred static networks with the results obtained from comparable inference algorithms such as ARACNE, CLR or LASSO, they were able to show that already the introduction of TFBS knowledge yielded to better performance. Moreover, they showed that an additional soft-integration of prior-knowledge further increased the reliability of the inferred gene-to-gene relationships.

### Network inference using ExTILAR

For the network inference, the measured expression profiles for the 22 DETFs as well as the mean gene expression profile for each cluster were scaled to an absolute maximum value of 1. Linearly interpolated data was added to provide equidistant measurements (*Δt*=1 hour). An exponentially decreasing input function was defined to model the change of culture medium.

**Figure 4:**
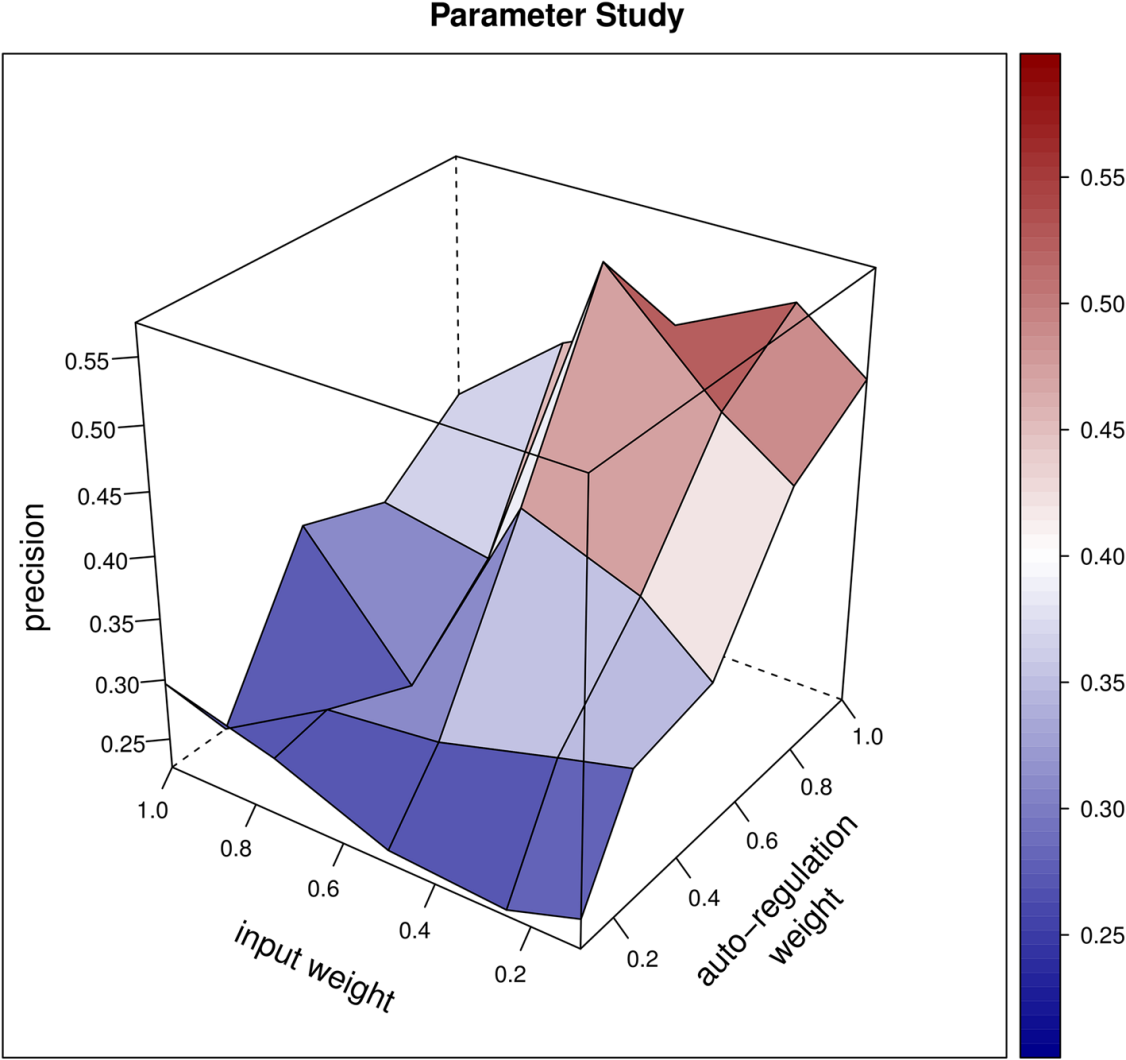
**Results of the parameter study optimizing the parameter values for the input weight and the auto-regulation weight.** Outlined is the ratio of the number of included prior-knowledge relations to the total number of inferred relations excluding the input-to-gene relations (precision). Based on this result and whether or not numerical simulation of the inferred network led to dynamics comparable to the observed ones, the auto-regulation weight was set to 0.75 while the input weight was set to 0.5.

**Figure 5:**
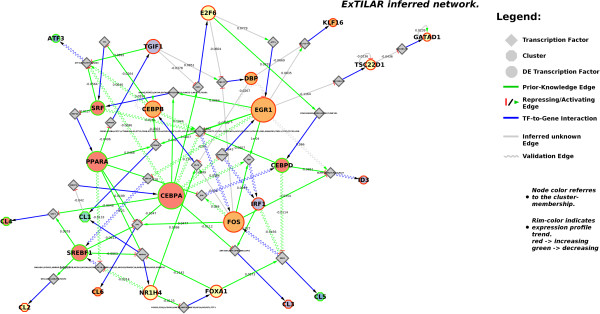
**Transcription factor network describing the cellular response of murine primary hepatocytes to the addition of fresh medium.** The ExTILAR-inferred transcription factor network consists of 3 types of nodes differentiated by their shape, the target DETFs (circle), the regulating transcription factors (diamond) and clusters (octagon). The color of the nodes denote for the corresponding cluster membership, while the rim color reflects the general tendency of the expression profile (increasing: red; decreasing: green). The size of the nodes corresponds to the number of outgoing edges (higher numbers equal more outgoing edges) and highlights DETFs with a hub-like role. TF-to-gene interactions are outlined using blue edges. Inferred gene-to-TF edges are either green or gray, depending on whether they are supported by prior-knowledge (green) or not (gray). The regulating function of these edges is reflected by the target arrow. A red bar denotes for repression while a green arrow represents activation. Waved edges represent inferred direct gene-to-gene relations which were found in literature. As this information was not used during the inference, the presence of these relations within the inferred network supports the validity of the constructed TFN

**Figure 6:**
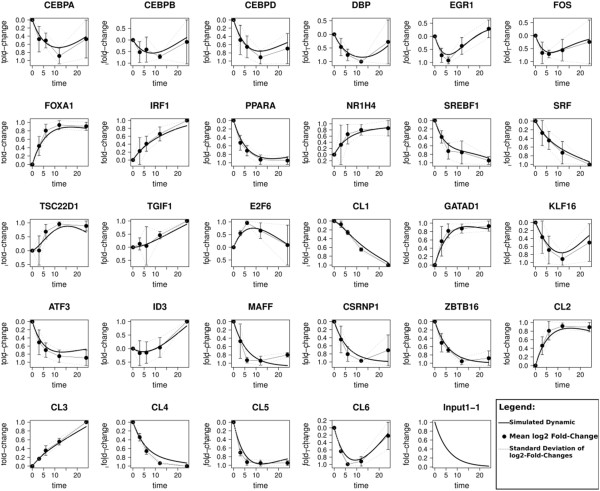
**Measured and simulated expression profiles.** The dots represent the measured mean log2-FC of the three replicates and the error-bars denotes for the standard deviation of the log2-FCs. The simulation results (solid lines) show the fit of the model to the measured data. The simulated dynamics are always close to the mean log2-FC and mostly within the bounds of the standard deviation.

In an initial parameter study the auto-regulation weight and the input weight were tuned by testing 25 combinations of these two parameters. The best results in terms of quality of the fit (deviation from the measured data, RSS), number of included prior-knowledge edges and number of the total edges was found when using an input weight of 0.5 and an auto-regulation weight of 0.75 (Figure 4). The delta parameter (*δ* of equation 7) which regulates the integration of prior-knowledge was set to 0.5. This setting, which corresponds to a moderate knowledge integration ensured that the prior-knowledge is not the driving force for determining the structure but is still respected. In the second analysis we tested the influence of the delta parameter on the quality of the predicted network in terms of prior-knowledge integration, the total number of inferred edges and the fit of the simulation to the measured data. Seven networks with decreasing delta value (1, 0.75, 0.5, 0.25, 0.1, 0.05 and 0.01) were inferred and the resulting networks were compared. Table 2 shows how the prior-knowledge is soft-integrated into the network with decreasing delta and highlight that a delta value of 0.1 offers the best balance between a high precision and a low RSS. A further decrease of delta (like 0.05 and 0.01) leads to over-fitting of the network to the prior knowledge which results in a strong decrease of quality of the simulated kinetics to the measured gene expression profiles (strong increase of RSS). The ExTILAR-inferred network (subsequently referred to as TFN) using these parameters is outlined in Figure 5 (for Cytoscape session see Additional file 4), the simulated gene expression dynamics are plotted in Figure 6. In Figure 5, the input node and all input-to-gene edges were removed for better visualization. The input-to-gene edges and their weights are listed in Table 3.

**Table 2:** Knowledge analysis results

**Delta**	**# of**	**# prior-knowledge**	**Precision**	**RSS**
	**edges**	**edges**		
1	56	5	0.0893	29.07801
0.75	54	12	0.2223	23.07626
0.5	47	27	0.5744	23.62479
0.25	49	25	0.5102	25.50705
**0.1**	**64**	**47**	**0.7344**	**23.79333**
0.05	81	60	0.7407	69.28333
0.01	119	77	0.6471	14233.39749

Results of the knowledge analysis using 7 different delta values (Delta) showing the total number of inferred relations (# of edges) excluding the input-to-gene edges, the number of integrated prior-knowledge gene-to-TF relations (# prior-knowledge edges), their ratio (Precision) and the deviation of the simulated data to the measured ones (the RSS as defined in equation 5).

**Table 3:** Inferred input-to-gene edges of the GRN

**Network nodes**	**Input-to-gene weight**
Egr1	-0.294
Fos	-0.257
Cl6	-0.242
Cl5	-0.241
E2f6	0.223
Dbp	-0.184
Cl2	0.184
Gatad1	0.184
Atf3	-0.181
Maff	-0.179
Ppara	-0.178
Foxa1	0.178
Cebpd	-0.174
Srebf1	-0.171
Csrnp1	-0.168
Zbtb16	-0.167
Cebpb	-0.159
Cebpa	-0.156
Cl4	-0.148
Nr1h4	0.136
Klf16	-0.132
Srf	-0.098
Irf1	0.088
Cl3	0.067
Id3	-0.051
Tsc22d1	0.034
Cl1	-0.025
Tgif1	0

Inferred input-to-gene edges which were removed from Figure 5 for better visualization. The network nodes (DETFs and clusters) are ordered according to their absolute input-to-gene weight.

### Network interpretation

The inferred TFN (Figure 5) is composed of clusters, DETFs and TFs. The clusters can be seen as functional modules representing biological processes the enclosed genes are involved in while the DETFs resemble measured genes for which the corresponding protein is known to exhibit transcription factor activity. The TFs are bridging elements that connect clusters and DETFs among each other. The ExTILAR inferred TFN consists of 91 inferred edges, four auto-regulatory edges, 27 input-to-gene edges (Table 3) and 60 gene-to-TF edges. 47 out of the 60 gene-to-TF edges are supported by prior-knowledge, which was given for the inference (green edges). The 13 remaining edges are predicted, novel interactions (gray edges). 15 relations of the extracted validation knowledge were found to be integrated in the network (waved edges). The negative auto-regulation edge of Egr1 however was found to be contrary to literature knowledge from smooth muscle cells [42].

In Figure 5, the node size of the modeled DETFs is determined by the number of outgoing relations. Notably, most of the DETFs have a low outdegree while only a few of them are highly connected. This observation is an important structural property which was found to be common for biological networks. In the TFN, this hub-like role is accomplished by Egr1 and Cebpa. They are highly connected to DETFs associated with diverse biological roles. Sorting the absolute weights of the input-to-gene relations (Table 3) reveals that Egr1 has the highest absolute weight (-0.294) just before Fos(-0.257), Cl6 (-0.242), Cl5 (-0.241) and E2f6 (0.223). Cebpa appears only at the 18th position (-0.156). This data shows that, according to the network, Egr1 but not Cebpa is one of the DETFs initially affected by the exchange of culture medium. This finding in the network is supported by current literature knowledge which identifies especially Egr1 as a distributing TF rather then a direct effector of physiological changes [43]. The DETFs of the TFN can roughly be divided into 2 groups describing the main biological functions observed as a response upon the exchange of the culture medium. The first group contains genes which are known to affect metabolic processes during the second 24 h cultivation period of primary mouse hepatocytes (Cebpa, Dbp, Foxa1, Nr1h4, Ppara, Srebf1, Srf, Tgif1). The second group consists of DETFs that can be associated with proliferation and regulation of the cell cycle (Atf3, Cebpb, Cebpd, Dbp, E2f6, Fos, Irf1, and Tsc22d1).

#### Regulation of metabolic processes

Regulation of metabolic processes is mainly exerted by Cebpa, Foxa1, Nr1h4, Ppara, Srf, Srebf1 and Tgif1. Of these DETFs, Cebpa plays a distributing role within this group. This is consistent with literature as the TF was described to be an important regulator of the energy metabolism [44]. The connection to cluster 4 further highlights the importance of Cebpa for processes such as the lipid and glucose metabolism [45-47]. Cluster 4 genes are significantly over-represented in these biological processes. Interestingly, Srebf1, Nr1h4 and Foxa1 are three genes which were found to be interconnected in two double negative loops. The first loop between Srebf1 and Nr1h4 is partially supported by validation knowledge as Nr1h4 is known to inhibit Srebf1 expression [48]. To our knowledge, they have not been considered in a loop jet. Srebf1 is affecting the lipid metabolism [49-51] and was found to be affected by the feeding regime [52-54]. Controversially to the experimental *in vitro* data given here, *in vivo* experiments showed that the TF is decreased during fastening and increased upon re-feeding [51]. Like Srebf1, Nr1h4 is also important for the lipid metabolism [55,56] and glucose homeostasis [57-59]. Additionally, the TF was found to regulate the bile acid metabolism and the metabolism of xenobiotics [60-63]. Interestingly, Nr1h4 is thought to modulate the fasting-re-feeding transition in mice [58].

The second loop, which is supported by prior-knowledge regarding the gene-to-TF interactions is formed by Foxa1 and Nr1h4. Foxa1 is also associated with metabolic processes and plays a central role in the glucose homeostasis [64-67].

Loops, where both edges are negatively regulating can exert a switch like function. This can lead to interesting biological features such as bistability of the system.

Tgif1 is one of the DETFs of which less is known so far. This TF was found to repress transcription of RXR and LXR target genes [68-71]. Both of these nuclear receptors are known to play important roles in the regulation of diverse metabolic functions such as the lipid and glucose metabolism. Interestingly, expression of Tgif1 is negatively related to the expression of Atf3, a TF that is associated to cell cycle regulation and proliferation as described in the next section. Therefore, Tgif1 might play a greater role then currently known, affecting metabolic as well as proliferative processes within hepatocytes.

#### Proliferation and regulation of the cell cycle

Hepatocytes remain in the quiescent G0 phase in the liver under normal conditions. Events that lead to the loss of liver mass result in the release of cytokines and the subsequent activation of TFs that prime the hepatocytes for proliferation. Zellmer *et al.*[28] showed that hepatocytes, cultured on collagen monolayers are primed through a cytokine independent activation of MAPK signaling within 24 hours after isolation. They identified Etf, E2f1 and Sp1 as having a potentially pronounced role in mediation of the proliferative effect within the first 24 hours after isolation. Since the present study uses the data of Zellmer *et al.* but focuses on the second phase of the experiment (24 hours after the exchange of culture medium), the aftereffects of the proliferation initiation and cell cycle regulation were monitored as well.

Among the DETFs modeled in the inferred network, regulation of proliferation and the cell cycle is mainly exerted by Atf3, Cebpb, Cebpd, Dbp, E2f6, Fos, Irf1 and Tsc22d1. Within this group, Fos is highly connected and regulated by seven other DETFs including Egr1. Moreover, Fos was found to have the second largest input-to-gene weight. This central function is supported by the finding that Fos expression is a pre-requisite for the reentry of quiescent cells into the cell cycle [72]. In the TFN, Fos is positively regulating Irf1 and Cebpd, both of which were found to be involved in proliferation and the cell cycle [73-75]. Interestingly, these two TFs can be found to negatively regulate Fos expression in turn. Irf1 is a gene for which the expression levels were found to be lowered after serum induced growth of serum starved cells in G0, and increased before and during the S phase [73]. Altogether, the decreasing expression profile of Fos and the continuously increasing expression profile of Irf1 supports the finding by Zellmer *et al.* that induction of proliferation happens in the first 24 hours after isolation. During the second phase however, the hepatocytes seem to be in the transition towards, or already in the S phase. The increasing expression profile of E2f6 is concordant with the identification of E2f1 as an important TF in the proliferation process. E2f6 was identified to repress E2f-activated transcription during the S phase, therefore balancing cell cycle control of E2f-family members such as E2f1 [76].

Id3, Atf3 and Tsc22d1 are rather terminal nodes within the inferred network and are (beside Tsc22d1) not regulating any other DETFs. This could be due to the missing available prior-knowledge regarding these DETFs. Atf3 is a transcriptional repressor that was found to delay cell cycle progression by slowing down the transition of the cell from G1 to S phase. It was shown that the TF also mediates positive and negative effects on proliferation [77,78]. Tsc22d1 is rather poorly investigated. Knock-down experiments of Tsc22d1 might reveal important functions associated to cell cycle control and proliferation in hepatocytes.

### Experimental validation

For experimental validation, we were interested whether or not a relation between Tgif1 and Atf3 expression exists. It is known that the two TFs are related as Atf3 has a promoter binding site for Smad3 [79]. Tgif1 was reported to act as a corepressor by binding to the activated Smad-complex (Smad2 and Smad3 can be bound) [70,80]. Altogether, this suggests that there might be a causal relation between Tgif1 expression and Atf3 expression in hepatocytes. However, to our best knowledge, a relation between these two TFs has not been shown so far.

To investigate the effect that a Tgif1 knock-down might have on the system, we performed an *in silico* knock-down using the inferred TFN (Additional file 5). Therefore, the logarithmized expression profile of Tgif1 was replaced by a linear decreasing function ranging from 0 at the 0h time point to -1 at the 24 h time point. Simulation of the network predicted an increase in Atf3, Cebpb and Klf16, and a decrease in Dbp. Analysis of the network shows that the increase in Cebpb and Klf16 both result from the decrease of Dbp, whereas the predicted increase in Atf3 is a result of the loss of repression by Tgif1 and the increase in Cebpb. Therefore, we also looked at Dbp expression, as this TF is directly regulated by Tgif1 in the network.

**Figure 7:**
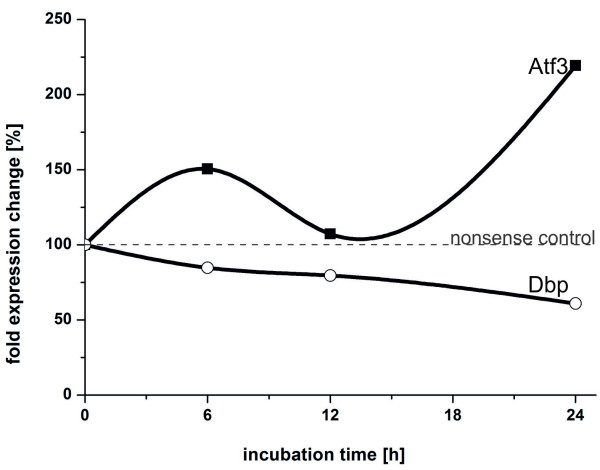
**Expression profiles of Atf3 and Dbp in response to the siRNA-mediated knock-down of Tgif1.** epatocytes were cultured for 24 hours and then transfected with siRNA against Tgif1 (referred to as zero time point) as described in Methods. After further incubation for 24 hours, RNA was extracted and expression levels of Atf3 (black squares) and Dbp (open circles) were determined by qRT-PCR.

For validation, a real siRNA-mediated knockdown of Tgif1 in cultured hepatocytes was carried out. At 6, 12 and 24 hours after transfection of the Tgif1 siRNA, expression levels of Atf3 and Dbp were measured using quantitative real time PCR (qRT-PCR).An unambiguous upregulation of Atf3 and a downregulation of Dbp were detected (Figure 7). Regarding the inhibition of Atf3 expression, this indicates that Tgif1 does not only play a role in altering metabolic processes such as lipid metabolism but might also positively affect hepatic proliferation and cell cycle regulation. The exact mechanism of repression however remains to be resolved, as three different modes of repression are currently known [80]. The downregulation of Dbp, although less severe, is of strong interest as it was shown that the expression of important cytochrome P450 family members is partially under the regulation of Dbp [81].

## Conclusions

In this work, the linear model of the recently published network inference algorithm TILAR was extended to infer ODE based network models. With this approach, the ExTILAR algorithm combines the benefits of the regression based TILAR (low computational costs, large network size processable, incorporation of various knowledge sources, partial separation of network structure identification and parameter estimation) with the possibilities that ODE based models offer (*in silico* simulations of the response to external perturbations, re-use in other models, possibility of integration into multi-scale modeling). Using a 5-node network to create *in silico* data we were able to show that ExTILAR and Net*Gene*rator inferred networks are of high quality with a performance advantage of ExTILAR over the Net*Gene*rator (Additional file 6). To make the algorithm easily accessible to the scientific community, we implemented ExTILAR in R. Together with the additional material, ExTILAR can be downloaded from http://www.hki-jena.de/index.php/0/2/490.

Applying the algorithm to biological data, we were able to present a TFN that models the main biological processes induced in hepatocytes upon culture medium exchange. We highlighted two possible regulatory loops between Srebf1, Nr1h4 and Foxa1. The function of these interesting network motifs will be motivation for further studies. Using a knock-down experiment read out by qRT-PCR, the biological relevance of the inferred network was shown by the validation of two hypothesized relations between Tgif1 and Atf3, and between Tgif1 and Dbp. Thereby, we detected new, potential functions of Tgif1 and further highlight the TF’s importance in the hepatic transcription factor network. Although the exact mechanism of regulation remains to be clarified, this example highlights how ExTILAR can be successfully used combining various prior-knowledge sources to infer biologically relevant, data supported regulatory networks.

## Methods

All analysis were performed using the biological data analysis package Bioconductor [82] for the statistical programming language R [83].

### Microarray pre-processing and gene filtering

Analysis of Affymetrix microarrays involves the initial annotation of the probe sets of the chips. A custom chip definition file is used to map the probes on the microarray to a genomic sequence and thus, to the transcript of a certain gene. However, it is well known that a large number of probe sets includes probes which match multiple transcripts and also probes which do not match any transcript [84]. Therefore, the custom chip definition file from Brainarray (Molecular and Behavioral Neuroscience Institute, University of Michigan) [29] based on Entrez-IDs was used for this analysis to obtain the gene expression intensity levels.

Detection calls of the raw data were obtained and used as an additional filter to remove uncertain probe sets. The method is used to remove transcripts for which the expression level is below the threshold of detection. This is described in detail in the Affymetrix Statistical Algorithms Description Document [32].

The mas5calls function of the affy package [85] was used with default settings to compute the detection calls. Probe sets declared as present or marginal in less then 80 percent of the analyzed microarrays were removed from the data set.

RMA [30] was used for pre-processing the data. This involved RMA background correction, quantile normalization and summarizing.

The pre-processed dataset was analyzed for DEGs using the two-fold criterion. A gene was called differentially expressed if its mean expression profile exhibited an absolute log2-FC of 1 or greater with respect to the 0 hour sample.

### Clustering and identification of over-represented TFBSs

The data was prepared for clustering by scaling each mean expression profile to the absolute maximum fold-change value of 1. The clustering algorithm and the number of clusters was determined by using the clValid package for R [86]. In total, nine clustering algorithms (hierarchical clustering, k-means, diana, fanny, SOM, PAM, SOTA, clara and model) were compared for 2 to 14 possible clusters regarding three internal validation measures (Dunn index [87], average silhouette width [88] and connectivity [89]) and stability validation measures (average proportion of non-overlap, average distance and average distance between means). These measures are outlined in detail in [86]. The SOTA algorithm was found to perform best using six clusters. The cluster enrichment analysis was performed using the GOStats package for R [33] based on the org.Mm.eg.db package [90].

oPOSSUM was used to search for over-represented TFBS among the genes of each cluster [37,38]. The top 14 TFBSs (ordered by the Z-Score) identified using a promoter region of 2000 base pairs upstream to the transcription start site were selected if they achieved a Z-Score of at least 4.8.

### ExTILAR GRN inference

The log2-FC profiles for the genes as well as the mean cluster log2-FC profiles were standardized to a maximum absolute log2-FC of 1. To obtain equidistant measurements for the regression based on difference equations, missing measurements were added using linear interpolation. An exponential decreasing input function was defined. This choice is based on the assumption that the change of culture medium is an initially strong stimulus that the cells adapt to. Over time, the stimulus becomes less severe as the effects induced in response to the stimulus become the dominating stimulus. Also, the supplied nutrients are consumed by all cells in the culture and thus, are decreased. However, together with the extracted TF-to-gene relations, the regression matrix was constructed according to equation 9. LARS was used to select and estimate the variables and calculate the *Cp*. As outlined in the original TILAR publication, we selected the model that minimizes the *Cp* statistic as sparse networks are favored [18]. A stepwise forward selection procedure was chosen to optimize the model structure. Starting from a network using no TF-to-gene relation, this procedure iteratively adds the edges that minimize the *RSS* of the inferred network compared to the *RSS* of the inferred network from the previous iteration. This iterative addition of TF-to-gene relations is stops if (i) there are no more TF-to-gene relations to add, or (ii) the *RSS* of the previous iteration is not undercuted. After the last iteration, the final network model was selected using the Pm measure described in the next section. We then performed an OLS fit of the final model using only the selected variables. Therefore, variable selection was performed using LARS but the actual estimation of the coefficient was obtained by a OLS fit. For details see the original TILAR publication [18].

### Model selection

Regardless of the implementation of LASSO used, the result is always a set of models with a differing number of variables (regression coefficients) and their estimates. The user has to apply a criterion to find a model with good quality. The quality of the network selection is always a trade-off between the data-fit and the number of parameters used in the network. One way to define the quality of a model is how well a model fits to the measured data, disregarding the number of parameters used. This is described by minimization of the *RSS*, which is defined in equation 5. Models, which are selected using the *RSS* criterion tend to include lots of parameters. This often results in a good fit but to the expense of interpretability due to a high number of edges in the network.

Another model selection criterion is the Mallows Coefficient *Cp*[91]. The *Cp* is penalizing model complexity by considering the *RSS* with respect to the number *p* of variables used.

$$\mathrm{Cp}=\frac{\mathrm{RS}S_{p}}{S^{2}}-M^{\prime \prime }+2p\;\text{with}\;S=\frac{\mathrm{RS}S_{p_{\max}}}{p_{\max}}$$

where *RSS*_*p*_ is the residual sum of squares of the model with *p* (*p*=1…*N*^*′′*^) variables and $\left(\mathrm{RS}S_{p_{\max}}\right)$ denotes for the mean full model *RSS* using all variables (*p*_*max*_=*N*^*′′*^).

The *Pm*is a third measure which is adding the scaled number of used regression coefficients *p* to the weighted (*α* with *α*∈*R* ; *α*≥1), scaled *RSS*.

$$\mathrm{Pm}=\alpha \frac{\mathrm{RS}S_{p}}{\mathrm{RS}S_{\max}}+\frac{p}{p_{\max}}$$

The model which minimizes the *Pm*is selected, as the inclusion of more variables into the model does not lead to a significant decrease of the *RSS*. The decrease is significant if the *RSS* diminishes faster then the variable size is increasing. Adjusting alpha to higher values increases the number of edges included in the model.

### Experimental procedure Tgif1-knock-down

#### Hepatocyte isolation, cultivation and transfection

Primary hepatocytes from C57BL/6-N mice were isolated by collagenase perfusion of the liver according to Gebhardt et al., 2003 [92]. Hepatocytes were suspended in Williams Medium E containing 10% fetal calf serum, 0.1 *μ*M Dexametasone, 2mM Glutamine and Penicillin/Streptomycin mix [28,93], and were plated onto 12-well plates pre-coated with collagen type I (0.1 Mio cells/well, sample replicates of three wells). Cells were incubated at 37°C and 5% CO_2_. After 2 hours, the cells were cultured with a serum-free medium for further 22 hours.

After 24 hours total cultivation time, the serum-free medium was renewed and chemically synthesized siRNA for Tgif1 (20 nmol) was transfected with INTERFERin^TM^ purchased from Peqlab (Erlangen, Germany) according to the manufacturer’s instructions. Tgif1-specific siRNA (Gene Solution siRNA; target sequence CACCTACAGTCTAATGAGTAA) and the respective scrambled control siRNA was purchased from Qiagen (Hilden, Germany). The cells were incubated with the siRNA for additional 6, 12 and 24 hours. Total RNA from hepatocytes was isolated with RNeasy plus Mini Kit (Qiagen, Hilden, Germany) from three wells and pooled.

#### Quantitative qRT-PCR

RNA was reverse transcribed using oligo(dt) primers and IM Promm II reverse transcriptase (Promega, Mannheim, Germany). The levels of the mRNA transcripts for Atf3, Dbp and *β*-actin as housekeeping gene were determined using gene-specific primers (Table 4). qRT-PCR measurements were carried out in duplicate using the Light Cycler® 2.0 Instrument and the LightCycler® FastStart DNA Master PLUS SYBR Green I (Roche, Grenzach-Wyhlen, Germany) or the Rotor Gene 6000® real-time PCR cycler and SYBR Green I (Qiagen, Hilden, Germany). The absolute quantitative analysis of the target genes were normalized to *β*-actin.

**Table 4:** Primers used for qRT-PCR analyses

**Gene**	**Primer forward 5’ → 3’**	**Primer reverse 5’ → 3’**
Tgif1	-GAAACCCCAGCTTCACCTCT-	-GCCAGATGCTGCAACAAG-
Atf3	-GCTGGAGTCAGTTACCGTCAA-	-CGCCTCCTTTTCCTCTCAT-
Dbp	-CTTTTGACCCTCGGAGACAC-	-TGGCTGCTTCATTGTTCTTG-
*β*-Actin	-CATCCGTAAAGACCTCTATGCCAAC-	-ATGGAGCCACCGATCCACA-

## Abbreviations

DETF: Differentially expressed genes for which the corresponding protein is known to exhibit transcription factor activity; GRN: Gene regulatory network; TFN: Transcription factor network; LARS: Least angle regression; TILAR: TFBS-integrating LARS; ARACNE: Reverse engineering of accurate cellular networks; CLR: Context likelyhood of relatedness; TFBS: Transcription factor binding site; ExTILAR: Extended TILAR; TF: Transcription factor; RMA: Robust multi-array average; log2-FC: Log2 fold-change; SOTA: Self-organizing tree algorithm; DEG: Differentially expressed gene; OLS: Ordinary least squares; ODE: Ordinary differential equation; Cp: Mallows Cp coefficient; RSS: Residual sums of squares; Pm: Proposal measure; qRT-PCR: Quantitative real time polymerase chain reaction; Egr1: Early growth response 1; Srf: Serum response factor; Tsc22d1: Transforming growth factor-beta stimulated clone-22; Atf3: Activating transcription factor 3; E2f6: E2F transcription factor 6; Irf1: Interferon regulatory factor 1; Cebpa: CCAAT/enhancer binding protein alpha; Foxa1: Forkhead box A1; Tgif1: TGFB-induced factor homeobox 1; Dbp: D site albumin promoter binding protein; Fos: FBJ osteosarcoma oncogen; Id3: Inhibitor of DNA binding 3; Maff: V-maf musculoaponeurotic fibrosarcoma oncogene family, protein F (avian); Nr1h4: Nuclear receptor subfamily 1, group H, member 4; Klf16: Kruppel-like factor 16; Zbtb16: Zinc finger and BTB domain containing 16; Srebf1: Sterol regulatory element binding transcription factor 1; Cebpb: CCAAT/enhancer binding protein beta; Ppara: Peroxisome proliferator activated receptor alpha, Sp1: Trans-acting transcription factor 1; E2f1: E2F Transcription factor 1.

## Competing interests

The authors declare that they have no competing interests.

## Authors’ contributions

RGu and RGe directed the study. RGe, SZ, MMS and EM performed the experiments and collected the data. SV carried out the analyses and wrote the paper. Part of the source code is from the TILAR publication of Hecker et al. [18]. The implementation for the stepwise forward selection was done by JL and extended by SV to work within the ExTILAR algorithm. RGu and RGe assisted in interpretation of the results and, together with JL, WSH and AMB, contributed writing the paper. All authors read and approved the final manuscript.

## Supplementary Material

Additional file 1 **Detailed table of differentially expressed genes.** Table of the differentially expressed genes containing the corresponding BrainarrayID, EntrezID, GenSymbol, assigned cluster number and the mean expression values at each time point. Click here for file

Additional file 2 **Enrichment analysis results of the DEGs.** Details of the gene enrichment analysis using all differentially expressed genes. Click here for file

Additional file 3 **Enriched GO-terms and KEGG-pathways of the single clusters.** Significant GO biological process (GO-BP) terms and KEGG-pathway (KEGG) terms for each cluster returned by GOstats. Click here for file

Additional file 4 **Transcription factor network.** Cytoscape session of the transcription factor network shown in Figure 5. Click here for file

Additional file 5 ***In silico* knock-down simulation.** An image of the network simulation results of the *in silico* Tgif1 knock-down. Click here for file

Additional file 6 **Performance analysis of ExTILAR.** A performance analysis of ExTILAR using *in silico* data for a systematic inference. The results are compared to the results of the Net*Gene*rator algorithm. Click here for file
