# Supplementary material for: The extended TILAR approach: a novel tool for dynamic modeling of the transcription factor network regulating the adaption to in vitro cultivation of murine hepatocytes
Source: BMC Syst Biol. 2012 Nov 29;6:147. doi: 10.1186/1752-0509-6-147 (PMC3573979; doi:10.1186/1752-0509-6-147)
Supplement: Additional file 3 — Enriched GO-terms and KEGG-pathways of the single clusters. Significant GO biological process (GO-BP) terms and KEGG-pathway (KEGG) terms for each cluster returned by GOstats. [file 1752-0509-6-147-S3.doc]

| **Cluster** | **GO-BP** | **KEGG** |
| --- | --- | --- |
| 1 | - oxidation reduction proc.  - lipid metabolic proc.  - organic acid metabolic proc. | - retinol metabolism  - metabolism of xenobiotics by cytochrome P450  - linoleic acid metabolism  - arachidonic acid metabolism  - PPAR signaling |
| 2 | - translation | - ribosome  - oxidative phosphorylation |
| 3 | - oxidation-reduction process | - glutathione metabolism |
| 4 | - organic acid metabolic process  - ketone metabolic process  - cellular amino acid metabolic process  - lipid metabolic process | - glycine, serine and threonine metabolism |
| 5 | - | - |
| 6 | - response to stimulus | - |
