# Supplementary figures and images for: The extended TILAR approach: a novel tool for dynamic modeling of the transcription factor network regulating the adaption to in vitro cultivation of murine hepatocytes
Source: BMC Syst Biol. 2012 Nov 29;6:147. doi: 10.1186/1752-0509-6-147 (PMC3573979; doi:10.1186/1752-0509-6-147)

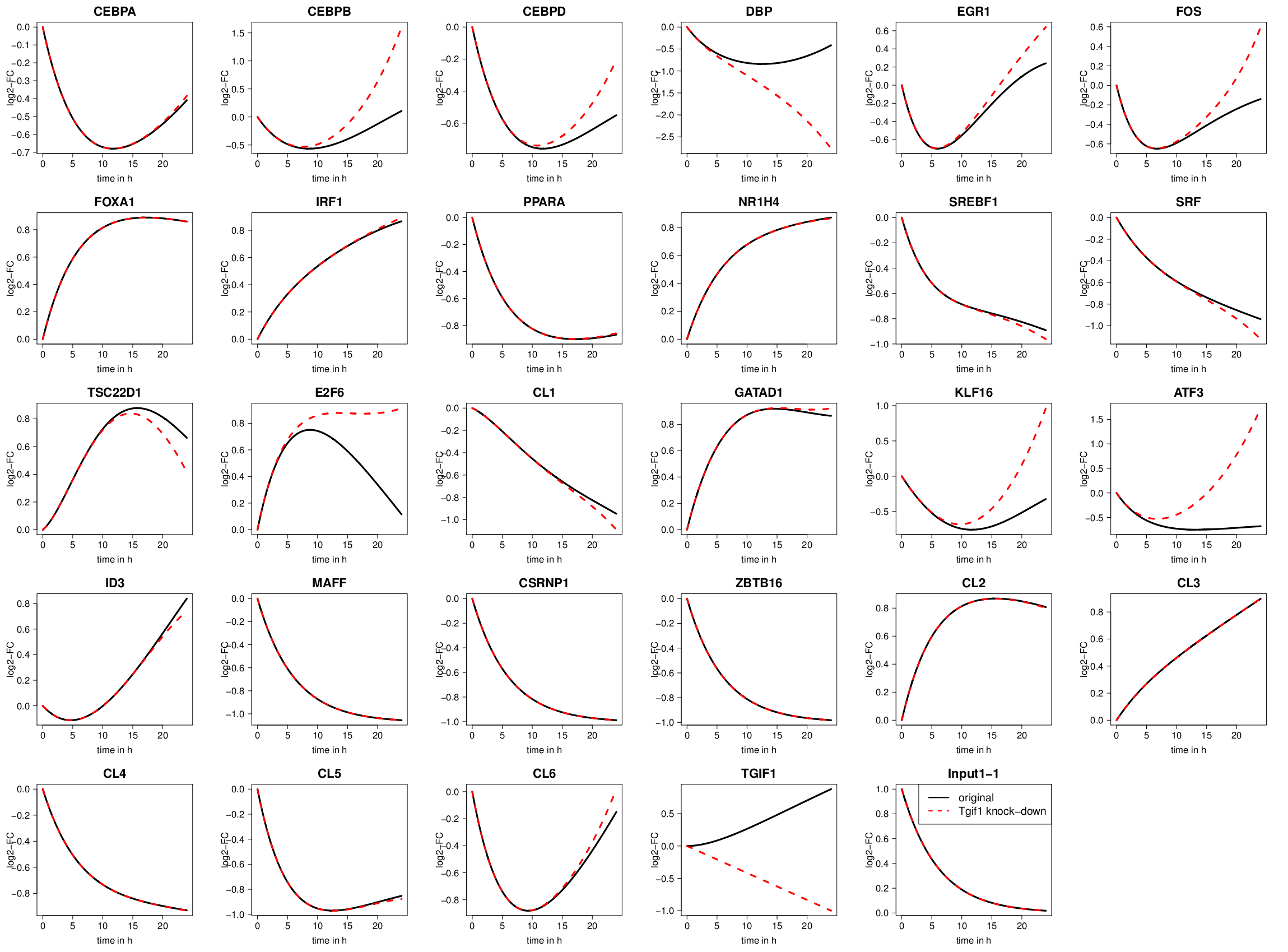

Supplement: Additional file 5 — In silico knock-down simulation. An image of the network simulation results of the in silico Tgif1 knock-down. [file 1752-0509-6-147-S5.png]
