# Supplementary material for: The extended TILAR approach: a novel tool for dynamic modeling of the transcription factor network regulating the adaption to in vitro cultivation of murine hepatocytes
Source: BMC Syst Biol. 2012 Nov 29;6:147. doi: 10.1186/1752-0509-6-147 (PMC3573979; doi:10.1186/1752-0509-6-147)
Supplement: Additional file 6 — Performance analysis of ExTILAR. A performance analysis of ExTILAR using in silico data for a systematic inference. The results are compared to the results of the NetGenerator algorithm. [file 1752-0509-6-147-S6.pdf]

The Extended TILAR Approach: A novel Tool for Dynamic Modeling of the Transcription Factor Network Regulating the Adaption to *in vitro* Cultivation of Murine Hepatocytes

- supplementary information -

Sebastian Vlaic*[1], Wolfgang Schmidt-Heck[1], Madlen Matz-Soja[2], Eugenia Marbach[2] , Jörg Linde[1], Anke Meyer-Baese[3], Sebastian Zellmer[2♯], Reinhard Guthke[1], Rolf Gebhardt[2]

[1]Leibniz Institute for Natural Product Research and Infection Biology – Hans-Knöll-Institute, Beutenbergstr. 11a, D-07745 Jena
[2]Institute for Biochemistry, Faculty of Medicine, University of Leipzig, Johannesallee 30, D-04103 Leipzig
[♯]current address: Federal Institute for Risk Assessment, Max-Dohrn Str. 8-10, D-10589 Berlin
[3]Department of Scientific Computing, Florida State University, Tallahassee, Florida 32310-4120, USA

Email: Sebastian Vlaic*- Sebastian.Vlaic@hki-jena.de; Wolfgang Schmidt-Heck - Wolfgang.Schmidt-Heck@hki-jena.de; Madlen Matz-Soja - madlen.matz@medizin.uni-leipzig.de; Eugenia Marbach - eugenia.marbach@medizin.uni-leipzig.de; Jörg Linde - Joerg.Linde@hki-jena.de; Anke Meyer-Baese - ameyerbaese@fsu.edu; Sebastian Zellmer - Sebastian.Zellmer@bfr.bund.de; Reinhard Guthke - Reinhard.Guthke@hki-jena.de; Rolf Gebhardt - Rolf.Gebhardt@medizin.uni-leipzig.de;

*Corresponding author

Performance evaluation of ExTILAR

To assess the performance of ExTILAR, the algorithm was systematically applied to *in silico* data obtained from a linear model. The ExTILAR inferred networks were then compared to the results of the published Net*Gene*rator algorithm [1, 2]. The workflow of the performance evaluation consists of essentially 5 steps (figure 1). First, a true, randomly generated gene regulatory network is chosen. Secondly, the generated network is simulated to obtain the expression data for the inference process. In a next step, ExTILAR is used in a parameter study to identify a optimal set of parameters for the inference. Subsequently, a perturbation analysis is performed to filter stable edges and finally, the resulting stable network is analyzed. This workflow was cycled 8 times while iteratively increasing the number of prior-knowledge edges.

In silico expression data generation

We used a 5 node network that was created using the Barabási-Albert model (figure 2). Therefore, this network has a node degree distribution which follows a power law with an exponent of -3 [3]. We simulated the network three times to obtain three replicate time series data consisting of five time points (0, 1, 2, 5, 10 hours). Gaussian noise (mean 0, standard deviation 0.1) was added to the data. This resulted in a data set of 5 genes, measured at 5 time points with three replicates. Finally, the measurements were scaled gene-wise to a maximum absolute expression value of 1. For both algorithms, a continuous input function was selected.

Network inference using ExTILAR

The advantage of the TILAR-based modeling concept is to incorporate transcription factor binding site (TFBS) knowledge into the inference process which initially excludes certain possible network structures. However, since there is no TFBS knowledge for this *in silico* network, each of the five genes has to be regulated by an artificial TF. This way, all network topologies are possible which leads to equal preconditions for both algorithms.

Prior to the inference, interpolated data was added to achieve equidistant measurements. This was done using the cubic splines function in R [4]. A parameter study testing 25 input parameter combinations for the auto-regulation weight and the input weight led to equally good results in terms of the deviation of the simulated data to the measurements and the number of edges included in the model. Therefore, the auto-regulation weight was set to 0.1 enforcing self-regulation which can also be interpreted as the sum of effects, such es auto-regulation and degradation rates. Input-to-gene edges were not favored by the algorithm as the input weight was set to 1. Stepwise forward selection was used to optimize the network topology and to find a structure that minimizes the residual sums of squares (RSS). Given the full set of solutions returned by LARS, the model that minimized the Pm was selected (for details see the Methods section).

Given the set of identified input parameters, inference of the GRN using ExTILAR was carried out. In the next step, the stability of the edges was determined performing a perturbation analysis. Gaussian distributed (mean 0, standard deviation 0.1) noise was added to the measured data, generating 1000 distinct data sets. Network inference using ExTILAR was performed for each generated data set and the results were used to create the final model. An edge was included into the final model, if it was inferred in at least a certain number of the networks. If prior-knowledge was given, this threshold was manually chosen depending on the number of integrated prior-knowledge edges, the total number of edges within the model and whether or not the predicted measurements were close to the measured ones.

This workflow was repeated 7 times with an iteratively increasing number of prior-knowledge edges. Figure 3 exemplary shows the ExTILAR inferred GRN for five prior-knowledge edges.

Network inference using Net*Gene*rator

The Net*Gene*rator is a successfully applied [5–7] network inference algorithm that produces networks based on ordinary differential equations. Since Net*Gene*rator is using only one time series, the mean of the three replicates was used for the inference process. At first, the parameters of the algorithm were optimized manually with respect to the average mean squared error, the number of prior-knowledge edges included in the model and the overall number of edges of the model. Using the optimized set of parameters, perturbation analysis and the filtering of stable edges were performed as described in the previous section.

This was repeated for an iteratively increasing number of prior-knowledge edges.

Quality measurements for networks

Given the inferred networks of the *in silico* data, different statistical measurements can be calculated to assess the quality of the results. To compute these measures, the edges have to be labeled as true positive (TP), true negative (TN), false negative (FN) or false positive (FP). There are two possibilities for an edge to be FP , either because there is truly no connection (FPn) or because there is a connection but with the wrong sign (FPs). Therefore, $FPn + FPs = FP$. Four quality measurements were calculated, the sensitivity (SE), the specificity (SP), the precision (PR) and the F-measure (FM). SE is a measure to evaluate the completeness of a solution. It computes the fraction of the identified TP of the total number of correct edges (equation 1). SP reports how well true negative edges can be identified by the algorithm (equation 2). Like the SE measure, PR reports the correctness of the solution. Here, the relation of the TP to the complete number of called edges in the solution ($TP + FP$) is calculated (equation 3). The F-measure is the weighted average of PR and SE enabling to evaluate both measures at the same time (equation 4).

$$SE = \frac{TP}{TP + FN + FPs} \quad (1)$$

$$SP = \frac{TN}{TN + FPn} \quad (2)$$

$$PR = \frac{TP}{TP + FPn + FPs} \quad (3)$$

$$FM = \frac{2 * PR * SE}{PR + SE} \quad (4)$$

Evaluation of the results

Since the gold standard is known, specificity, sensitivity, precision and the F-measure were calculated for the networks (table 1). The results were compared to the the ones of the Net*Gene*rator (figure 4). The results show that using a low number of prior-knowledge edges, ExTILAR tends to miss more TP edges than the Net*Gene*rator. This is indicated by the low SE values. More interesting is the constantly high number of SP which shows that if ExTILAR selects a edge, it is likely to be a TP edge. This shows that compared to Net*Gene*rator, ExTILAR infers networks with less edges. This relation is also reflected in the high PR values of the ExTILAR inferred networks. Comparing the overall performance using the FM shows that if prior-knowledge is used, ExTILAR slightly outperforms Net*Gene*rator. However, this can be a result of the parameter optimization as only a few of the available parameters for the Net*Gene*rator algorithm were optimized. A second reason could be that ExTILAR works on all three generated replicates at the same time while Net*Gene*rator has to rely on the mean values. Altogether, these results indicate that both algorithms are able to perform well on *in silico* data, with a increased performance of ExTILAR when prior-knowledge is available. However, it needs to be kept in mind that the main advantage of ExTILAR, namely the incorporation of TFBS knowledge was not tested here.

Conclusions

We evaluated the performance of ExTILAR by systematic inference of networks using *in silico* data. Comparing the results to the published Net*Gene*rator algorithm showed that ExTILAR leads to networks of equal quality in terms of data fit with a mostly stronger performance when prior-knowledge is available.

Abbreviations

GRN: gene regulatory network; LARS: least angle regression; TILAR: ExTILAR: extended TILAR; TF: transcription factor; TFBS: transcription factor binding site; log2-FC: log2 fold-change; RSS: residual sums of squares; TP: true positive; TN: true negative; FP: false positive; FN: false negative; FPs: false positive - wrong sign; FPn: false positive - not in the model; SE: sensitivity; SP: specificity; PR: precision; FM: F-measure

References

1. Guthke R, Möller U, Hoffmann M, Thies F, Töpfer S: **Dynamic network reconstruction from gene expression data applied to immune response during bacterial infection.** *Bioinformatics* 2005, **21**(8):1626–1634, [http://dx.doi.org/10.1093/bioinformatics/bti226].

2. Toepfer S, Guthke R, Driesch D, Woetzel D, Pfaff M: **The NetGenerator algorithm: reconstruction of gene regulatory networks.** *Lect Notes Bioinf* 2007, **4366**:119–130.

3. Barabasi, Albert: **Emergence of scaling in random networks**. *Science* 1999, **286**(5439):509–512.

4. Team RDC: *R: A Language and Environment for Statistical Computing.* R Foundation for Statistical Computing, Vienna, Austria 2011, [http://www.R-project.org/]. [ISBN 3-900051-07-0].

5. Linde J, Wilson D, Hube B, Guthke R: **Regulatory network modelling of iron acquisition by a fungal pathogen in contact with epithelial cells.** *BMC Syst Biol* 2010, **4**:148, [http://dx.doi.org/10.1186/1752-0509-4-148].

6. Linde J, Hortschansky P, Fazius E, Brakhage AA, Guthke R, Haas H: **Regulatory interactions for iron homeostasis in Aspergillus fumigatus inferred by a Systems Biology approach.** *BMC Syst Biol* 2012, **6**:6, [http://dx.doi.org/10.1186/1752-0509-6-6].

7. Tierney L, Linde J, Müller S, Brunke S, Molina JC, Hube B, Schöck U, Guthke R, Kuchler K: **An Interspecies Regulatory Network Inferred from Simultaneous RNA-seq of Candida albicans Invading Innate Immune Cells.** *Front Microbiol* 2012, **3**:85, [http://dx.doi.org/10.3389/fmicb.2012.00085].

Figures

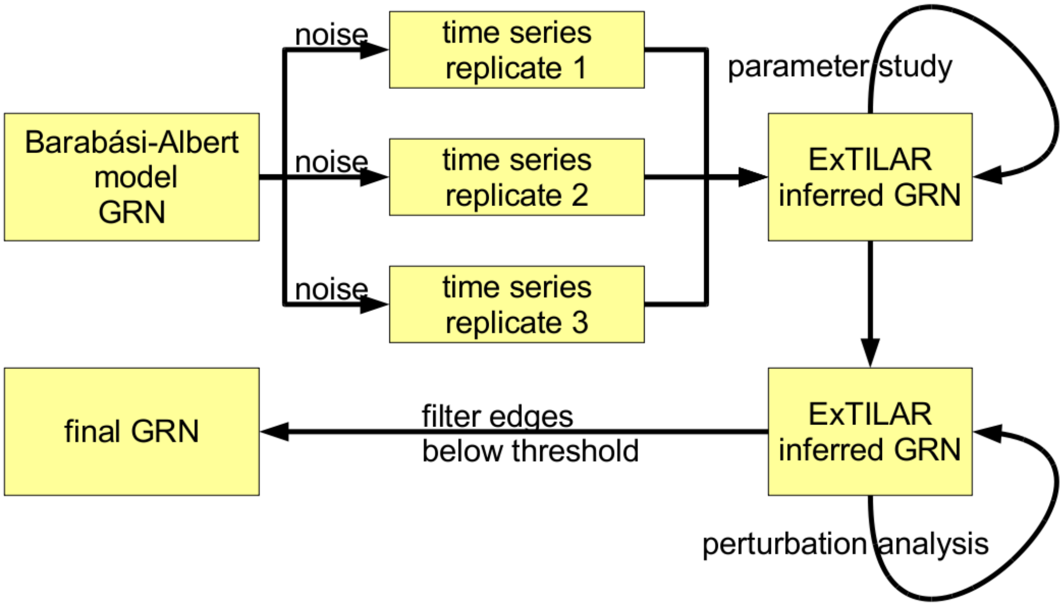

Figure 1 - Workflow of the performance evaluation of ExTILAR using *in silico* data.
The workflow of performance evaluation consists of 5 steps. (i) creation of an *in silico* true regulatory network, (ii) addition of noise to the measured values and generation of replicate measurements, (iii) identification of a good set of parameters for the inference process, (iv) removal of unstable edges by continuous re-inference of the network using perturbed data and (v) the final simulation.

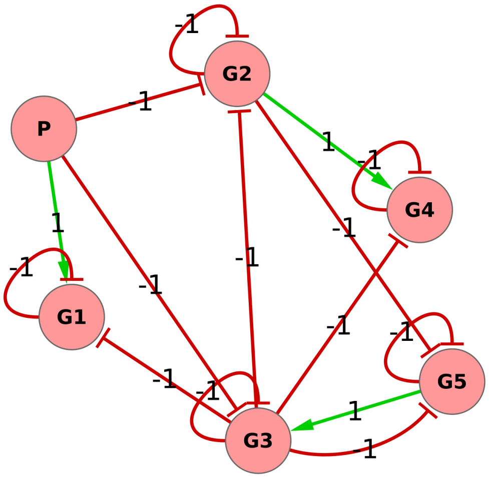

Figure 2 - In silico gene regulatory network to create expression data.
The gene regulatory network that was used to generate the *in silico* data. The nodes G1 to G5 represent the genes while the node P corresponds to the input perturbation which was chosen to be a continuous function of 1. The edge labels represent the weight of the edge with -1 representing a repressing edge while 1 denotes for a activating edge.

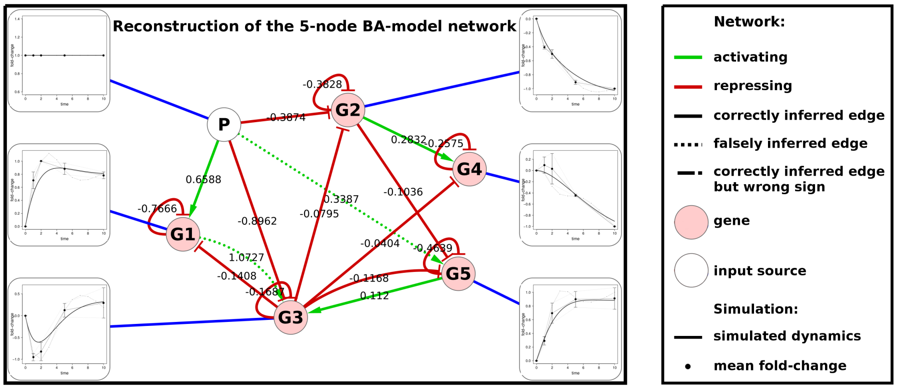

Figure 3 - BA-model test network and the inference results using ExTILAR with 5 edges of prior knowledge

Stable network inferred by ExTILAR using 5 prior knowledge edges. The plotted dynamics indicate a good fit to the data. In the network model, green edges are activating while red edges are inhibiting. The dotted edges are additional, false positive edges. The solid lines represent the simulated dynamics. Also indicated are the standard deviations for each measured time point. The dynamics of the genes follow the mean log2-FC without observable over-fitting but still mostly within the range of the standard deviation.

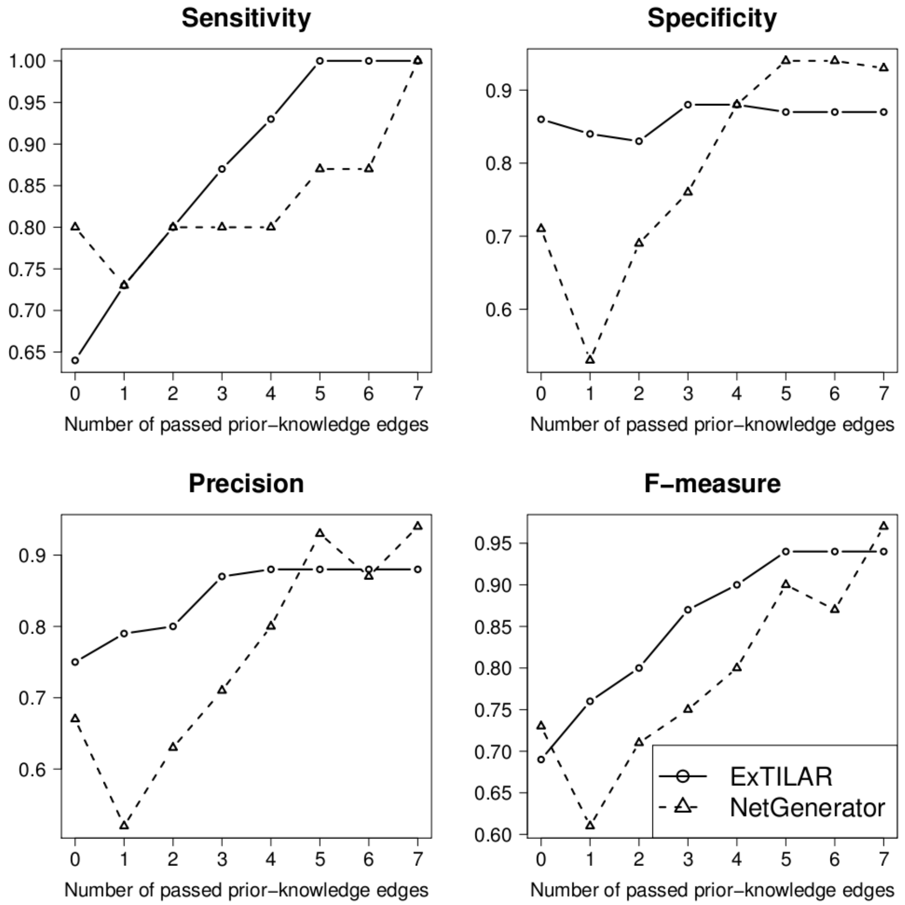

Figure 4 - Benchmarking results of ExTILAR and Net*Gene*rator.
Sensitivity, specificity, precision and the F-measure for the eight inferred networks. ExTILAR was found to mostly outperform Net*Gene*rator when prior-knowledge is available.

Tables

Table 1 - ExTILAR and NetGenerator: Summary of the network inference results.

Edges which were found to be true positive (TP), false positive with the wrong sign (FPs), false positives which are truly absent (FPn), false negative (FN) and true negative (TN). Given these values, quality measurements were calculated, namely sensitivity (SE), specificity (SP), precision (PR) and the F-measure (FM).

Algorithm	Knowledge	TP	FPs	FPn	FN	TN	SE	SP	PR	FM
ExTILAR	no	9	0	3	5	18	0.64	0.86	0.75	0.69
NetGenerator		12	1	5	2	12	0.80	0.71	0.67	0.73
ExTILAR	1 edge	11	0	3	4	16	0.73	0.84	0.79	0.76
NetGenerator		11	2	8	2	9	0.73	0.53	0.52	0.61
ExTILAR	2 edges	12	0	3	3	15	0.80	0.83	0.80	0.80
NetGenerator		12	2	5	1	11	0.80	0.69	0.63	0.71
ExTILAR	3 edges	13	0	2	2	15	0.87	0.88	0.87	0.87
NetGenerator		12	1	4	2	13	0.80	0.76	0.71	0.75
ExTILAR	4 edges	14	0	2	1	14	0.93	0.88	0.88	0.90
NetGenerator		12	1	2	2	15	0.80	0.88	0.80	0.80
ExTILAR	5 edges	15	0	2	0	13	1.00	0.87	0.88	0.94
NetGenerator		13	0	1	2	16	0.87	0.94	0.93	0.90
ExTILAR	6 edges	15	0	2	0	13	1.00	0.87	0.88	0.94
NetGenerator		13	1	1	1	15	0.87	0.94	0.87	0.87
ExTILAR	7 edges	15	0	2	0	13	1.00	0.87	0.88	0.94
NetGenerator		14	1	1	0	14	0.93	0.93	0.88	0.97
